# Mechanical Properties of Additive-Manufactured Composite-Based Resins for Permanent Indirect Restorations: A Scoping Review

**DOI:** 10.3390/ma17163951

**Published:** 2024-08-08

**Authors:** Giny Judith Pot, Patricia Anna Van Overschelde, Filip Keulemans, Cornelis Johannes Kleverlaan, João Paulo Mendes Tribst

**Affiliations:** 1 Department of Reconstructive Oral Care, Academic Centre for Dentistry Amsterdam (ACTA), Universiteit van Amsterdam and Vrije Universiteit, 1081 LA Amsterdam, The Netherlands; g.j.pot@student.acta.nl (G.J.P.); p.a.van.overschelde@student.acta.nl (P.A.V.O.); f.keulemans@acta.nl (F.K.); 2 Department of Dental Materials, Academic Centre for Dentistry Amsterdam (ACTA), Universiteit van Amsterdam and Vrije Universiteit, 1081 LA Amsterdam, The Netherlands; c.kleverlaan@acta.nl

**Keywords:** mechanical properties, 3D printing, dental materials, composite-based resin, permanent indirect restorations, fixed dental prosthesis, CAD/CAM, additive manufacturing

## Abstract

The introduction of 3D printing technology in dentistry has opened new treatment options. The ongoing development of different materials for these printing purposes has recently enabled the production of definitive indirect restorations via 3D printing. To identify relevant data, a systematic search was conducted in three databases, namely PubMed, Scopus, and Web of Science. Additionally, a manual search using individual search terms was performed. Only English, peer-reviewed articles that encompassed in vitro or in vivo research on the mechanical properties of 3D-printed composite materials were included, provided they met the predefined inclusion and exclusion criteria. After screening 1142 research articles, 14 primary studies were selected. The included studies mainly utilized digital light processing (DLP) technology, less commonly stereolithography (SLA), and once PolyJet printing technology. The material properties of various composite resins, such as VarseoSmile Crown Plus (VSC) and Crowntec (CT), were studied, including Vickers hardness, flexural strength, elastic modulus, compressive strength, tensile strength, fracture resistance, and wear. The studies aimed to compare the behavior of the tested additive composites to each other, conventional composites, and subtractive-manufactured materials. This scoping review examined the mechanical properties of composites used for 3D printing of definitive restorations. The aim was to provide a comprehensive overview of the current knowledge on this topic and identify any gaps for future research. The findings suggest that 3D-printed composites are not yet the first option for indirect restorations, due to their insufficient mechanical properties. Due to limited evidence, more research is needed in this area. Specifically, there is a need for clinical trials and long-term in vivo research.

## 1. Introduction

Digitalization has become more common in dentistry and is now an essential part of modern practice. Many dental practices are adopting intraoral scanning technology to capture patients’ oral situations digitally. Moreover, CAD/CAM (computer-aided design/computer-aided manufacturing) technology is being explored by dentists, dental technicians, and other stakeholders in the field [1,2]. This technology is based on data collection, data processing, and material production, and it has introduced various new processing techniques and materials [2,3]. The new materials include composite resins for permanent indirect restorations [4]. CAD/CAM technologies encompass both additive and subtractive production methods. Subtractive manufacturing in dentistry is utilized for milling a block of material into the desired shape. Additive manufacturing is better known as 3D printing, which is often referred to as the next revolution in dentistry and has a promising future [2,3,5].

The process of 3D printing involves constructing an object layer-by-layer from a raw material based on its virtual image. The virtual image is created from a 3D computer file of the object, a standard tessellation language (.STL) file, which is then digitally split into multiple layers. These layers are printed and fused [6,7]. Three-dimensional printing technologies are also known as “additive manufacturing” or ”rapid prototyping”. Among a variety of 3D printing techniques, stereolithography (SLA) and fused deposition modeling (FDM) are well-known forms [2,8]. Digital light processing (DLP) and SLA are two commonly used 3D printing techniques in dentistry. These methods are popular because they provide high accuracy, printing speed, and quality of printed surfaces, and are cost-effective. Moreover, they allow for more compact versions of the printers [8,9]. An SLA 3D printer works by using an ultraviolet laser light source to polymerize photosensitive resin. The laser draws a cross-section of the object to form a new layer, which is then polymerized. The build platform is submerged in the uncured printing fluid to cover the previous layer and the printing process is repeated. By repeating this process, the intended printed object is eventually created. The DLP printing technology is similar to SLA. The main difference between the two printing technologies is the light source. In DLP, the shape of the printed layer is projected onto the build platform using a projector [10].

Compared to traditional lost-wax techniques and computer-controlled milling, 3D printing offers several advantages in the production process [3,11]. With 3D printing, it is possible to produce a wide range of materials, including polymers, metals, and ceramics [2,5]. Other benefits of this technology include higher precision, faster production times, potential cost savings, improved workflow, reduced waste, decreased CO_2_ emissions, energy efficiency, and the ability to offer more personalized services [2,3,8,11].

**Figure 1:**
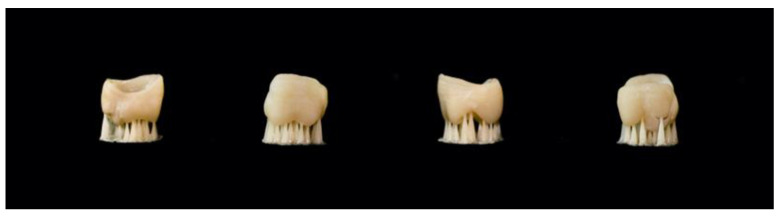
Crown printed with VarseoSmile Crown Plus, BEGO (mesial, buccal, distal, palatal).

Three-dimensional printers have become very useful in dentistry due to their ability to produce physical dental models, manufacture surgical guides for implantology, and create custom-made dental pieces for patients [1]. However, the effectiveness of 3D printing in dentistry largely depends on the materials used, which must meet certain mechanical, physical, and biological properties [5]. Innovative 3D printing techniques have brought new materials, including different polymers, into dental applications. Currently, 3D-printed polymers are being used in restorative and reconstructive dentistry to create dental models, occlusal splints, fixed and removable prosthetic devices, and temporary restorations. With its numerous advantages, 3D printing may eventually replace widely used CAD/CAM milling techniques in the field of dentistry [12]. The mechanical properties of a composite material are influenced by the composition and percentage of filler particles, which may lead to potential drawbacks. Besides the filler percentage, the color stability and aesthetics should also be considered when using 3D-printed composites for indirect restorations [13]. These composition and percentage of filler particles also determine the specific application of the material [14]. Currently, there are only a limited number of composite materials available to produce 3D-printed indirect permanent restorations [3,15]. It has been observed that the instructions for dental materials do not consistently use the term “composite”. Some materials are labeled as composites, such as Crowntec (Saremco Dental AG), while others are referred to as hybrid composites, such as VarseoSmile Crown Plus (Bego) (Figure 1).

The field of digital dentistry and chairside work could potentially be revolutionized with the introduction of new materials for producing permanent crowns and bridges. Although the materials for printed permanent indirect restorations hold great potential, they are still under active development. While there is a wide array of information about conventional composites in dentistry, there is limited information regarding commercially available 3D-printed composite resins and their mechanical properties for the manufacturing of permanent indirect restorations, such as crowns [16]. To address this, a scoping review was conducted to systematically gather literature on the mechanical properties of composite resin materials for the additive manufacturing of permanent indirect restorations. The research also aims to identify any information gaps that might exist.

The formulated research question is stated as follows: “What is the existing knowledge from the literature regarding the mechanical properties of 3D-printed composite resins for permanent indirect restorations?”. The research hypothesizes that the current composite resin materials used for 3D-printed indirect restorations do possess the required mechanical properties for a permanent indirect restoration.

## 2. Methods

### 2.1. Protocol

The protocol of this scoping review was made prospectively and is available online (https://osf.io/g5qzn/, accessed on 1 July 2024). The protocol used for this scoping review is based on the PRISMA extension for scoping reviews (PRISMA-ScR) by Tricco et al. (2018), which, in turn, examined and revised the existing protocol for systematic reviews to write a scoping review [17]. The PRISMA checklist is provided in the Supplementary Materials.

### 2.2. Research Question

What is known from the literature about the mechanical properties of 3D-printed composite resins for permanent indirect restorations?

### 2.3. Eligibility Criteria

#### 2.3.1. Inclusion Criteria

The studies that were included are peer-reviewed journal papers that were written in English. Both in vitro and in vivo studies that describe various mechanical properties of 3D-printed composite resins were included. These mechanical properties comprise, among others, flexural strength, Vickers hardness, wear resistance, surface wear, fatigue failure load, fracture load, toughness, and abrasion resistance. All articles had to be available in full text.

#### 2.3.2. Exclusion Criteria

The study focused solely on composite resin materials for 3D-printed permanent indirect restorations. Articles that did not discuss these materials, such as ceramics (zirconia and lithium disilicate), metals, and polyetheretherketone (PEEK), were not included in the study. Any studies that described the use of 3D printing techniques other than those for printing resin materials, such as SLM (selective laser melting), were excluded. Additionally, articles discussing temporary (provisional) restorations, removable prostheses, biomaterials, and non-dental applications such as craniofacial reconstruction were not relevant to the research question.

### 2.4. Information Source

To search for potentially relevant literature three databases were used: PubMed, Scopus, and Web of Science. The search strategy was developed by the researchers of this scoping review and further refined by the rest of the team. No limitations were added to the search strategy regarding publication. The most recent search was conducted on 22 February 2024, and is explained in Section 2.5, which follows.

### 2.5. Search

A search was conducted using the PubMed, Scopus, and Web of Science databases. The search strategy used MeSH terms and free-text terms for PubMed, title–abstract–keyword terms for Scopus, and ALL terms for Web of Science. All three search strategies had limitations.

#### 2.5.1. PubMed

The PubMed search, which was limited to articles in English, resulted in 278 articles. The search was as follows:

((printing, three-dimensional [MeSH Terms]) OR (Additive manufacturing) OR (Stereolithography) OR (Fused deposition modeling) OR (SLA) OR (FDM) OR (rapid prototyping) OR (digital light processing) OR (DLP) OR (digital light synthesis) OR (DLS)) AND ((Composite Resins[Mesh]) OR (dental polymers) OR (resin-based composites)) AND ((Mechanical Phenomena[Mesh]) OR (Mechanical Tests[Mesh]) OR (mechanical properties) OR (material strength) OR (abrasion resistance) OR (wear resistance) OR (surface wear) OR (Toughness) OR (fracture load) OR (fatigue failure load) OR (Stress)) NOT ((provisional) OR (interim) OR (Temporary)).

#### 2.5.2. Scopus

The Scopus search provided 370 results, of which 342 were articles, limited to English language and open-access articles. The search was as follows:

TITLE-ABS-KEY ((printing, AND three AND dimensional) OR (additive AND manufacturing) OR (stereolithography) OR (fused AND deposition AND modeling) OR (sla) OR (fdm) OR (rapid AND prototyping) OR (digital AND light AND processing) OR (dlp) OR (digital AND light AND synthesis) OR (dls)) AND TITLE-ABS-KEY ((composite AND resins) OR (dental AND polymers) OR (resin-based AND composites)) AND TITLE-ABS-KEY ((mechanical AND phenomena) OR (mechanical AND tests) OR (mechanical AND properties) OR (material AND strength) OR (abrasion AND resistance) OR (wear AND resistance) OR (surface AND wear) OR (toughness) OR (fracture AND load) OR (fatigue AND failure AND load) OR (stress)) AND NOT TITLE-ABS-KEY ((provisional) OR (interim) OR (temporary)) AND (LIMIT-TO (LANGUAGE, “English”)) AND (LIMIT-TO (OA, “all”)).

#### 2.5.3. Web of Science

The search conducted in Web of Science with limitations on language, open-access, and document type “articles” yielded 522 articles. The search was as follows:

ALL = ((printing, three dimensional) OR (Additive manufacturing) OR (Stereolithography) OR (Fused deposition modeling) OR (SLA) OR (FDM) OR (rapid prototyping) OR (digital light processing) OR (DLP) OR (digital light synthesis) OR (DLS)) AND ALL = ((Composite Resins) OR (dental polymers) OR (resin-based composites)) AND ALL = ((Mechanical Phenomena) OR (Mechanical Tests) OR (mechanical properties) OR (material strength) OR (abrasion resistance) OR (wear resistance) OR (surface wear) OR (Toughness) OR (fracture load) OR (fatigue failure load) OR (Stress)) NOT ALL = ((provisional) OR (interim) OR (Temporary)).

As part of the database search process, the search strings mentioned earlier were used. Additionally, a search in PubMed was carried out using individual search terms. The search terms ”Varseosmile” and ”Crowntec” were individually entered into the PubMed search bar. The search for ”Varseosmile” generated 17 articles, out of which 2 were selected for full-text screening based on their title and abstract. The term “Crowntec” produced 14 articles, of which 4 results were full-text screened. Ultimately, one article for “Varseosmile” and two articles for “Crowntec” were included based on the single search terms.

### 2.6. Selection of Sources of Evidence

**Figure 2:**
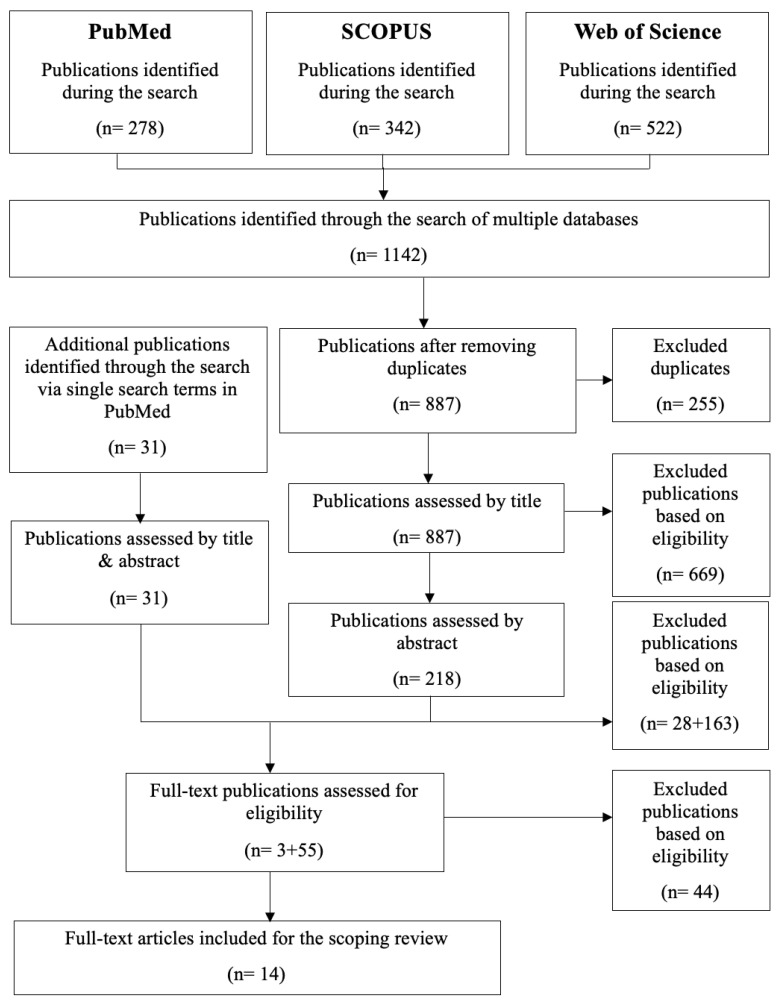
Methods flow chart.

The search strategies were applied to various databases. The resulting 1142 articles were imported into “Mendeley” (Version 2.114.1), a reference management software. Duplicate articles were identified using the software and 185 duplicates were removed. The remaining 957 articles were manually screened for remaining duplicates, resulting in an additional 70 duplicates being removed. After duplication screening, 887 articles were assessed based on their titles. Of these, 669 articles did not meet the inclusion and exclusion criteria. The remaining 218 articles were evaluated based on their abstracts for relevance. This resulted in the selection of 45 articles for full-text screening and 14 articles required review by a third party. Out of these 14 articles, the third party selected 10 articles for full-text screening, making the total number of articles for full-text screening 55, in which 12 articles were found to be usable. The final selection via the search strings and single search terms consisted of 14 articles (Figure 2). The screening process was carried out individually by two researchers, who then discussed any discrepancies and tried to reach a consensus. If they were unable to do so, a third party was consulted for further clarification.

### 2.7. Data Charting Process and Data Items

To answer a research question, relevant information must be collected from selected articles. This is achieved by preparing a table with important categories and subcategories. These categories and subcategories enable consistent data extraction from articles. To ensure that all researchers are aligned regarding the categories, each researcher individually formulated relevant information per category in a table from five articles. These independent tables were then compared for consistency. If any inconsistencies were found, they were discussed to clarify the intended meaning of the category in question. After individual data extraction, it was found that all researchers were aligned, and thus the full data extraction could begin. 

The selected articles’ data are categorized as shown below:

- Study characteristics:
  ○ Authors;
  ○ Year of publication.
- Materials:
  ○ Additive-manufacturing resin;
  ○ Subtractive-manufacturing resin;
  ○ Conventional-manufacturing resin;
  ○ Commercial name;
  ○ Sample size.
- Material manufacturer
- Material indication:
  ○ Temporary indirect restorations;
  ○ Permanent indirect restorations;
  ○ Direct restorations.
- Printer technology:
  ○ SLA;
  ○ DLP.
- 3D printing parameters:
  ○ Cleaning method;
  ○ Post-curing method;
  ○ Print orientation;
  ○ Print layer thickness.
- Measurements:
  ○ Mechanical properties;
  ○ Other properties.
- Geometry of the 3D-printed specimens:
  ○ Size/dimension;
  ○ Shape.
- Results.

### 2.8. Synthesis of Results

Fourteen articles were included in this study after an extensive search in the three databases. A summary of these articles is presented in Table 1, which provides an overview of the author, publication year, materials, manufacturer, material indication, printer technology, 3D printing parameters, measurements, geometry of the 3D-printed specimens, and results. Additionally, an attempt is made to describe the main data that emerged from the included articles.

The results are categorized based on material properties. Moreover, the factors that have a direct or indirect impact on these properties are explained. The material properties that are described include Vickers hardness, flexural strength, elastic modulus, fracture resistance, compression strength, tensile strength, and wear.

The researchers attempted to classify the evidence based on specific characteristics such as the composite used for 3D printing, the geometry of the specimens, and the material properties. This analytical approach allows for a more detailed representation of the various parameters relevant to the study that arose during the research. This scoping review aims to provide a comprehensive summary of the available scientific literature to achieve the set objectives.

## 3. Results

### 3.1. Mechanical Properties

#### 3.1.1. Vickers Hardness

Vickers hardness refers to the ability of a material to resist plastic deformation and is expressed as the Vickers hardness number (VHN) [7]. The hardness is measured by applying a specific force (newtons or grams) for a set period (in seconds) on the surface of the material using a diamond pyramid-shaped indenter [7,12,16,18].

According to a study conducted by Bora et al. in 2023, 3D-printed composites had a lower Vickers hardness compared to ceramic materials. Additionally, Borella et al. (2023) stated that the VHN varied significantly among different composite brands (*p* < 0.001). Among the brands tested, VarseoSmile Crown Plus (VSC) and Nanolab 3D (NNL) had a similar VHN (VSC 23.8 VHN; NNL 25.2 VHN). The VHN of VSC and NNL was higher than Resilab 3D Temp (RSL) (14.8 VHN) and NextDent Crown & Bridge MFH (MFH) (15.3 VHN) when printed at a layer thickness of 50 μm. When printed at a layer thickness of 100 μm, NNL had the highest VHN followed by VSC, while MFH and RSL had the lowest VHN. The study also found that the thickness of the printing layer affected the VHN not only among different brands but also within different material groups (*p* < 0.01) [7]. 

Additive-manufactured composites (Crowntec and Varseosmile Crown plus) had a lower hardness when compared to CAD/CAM-milled materials (*p* < 0.001), as found by Cakmak et al. (2023). The microhardness was measured after polishing, after brushing, and after coffee thermal cycling. CT had the lowest mean VHN (30.59 N/mm^2^, 29.74 N/mm^2^, 30.49 N/mm^2^, respectively). For VSC, the VHNs were 34.57 N/mm^2^, 33.26 N/mm^2^, and 32.47 N/mm^2^. The subtractive materials compared were Brilliant Crios (BC), Vita Enamic (VE), and Vita Mark II (VM). The VHNs of BC were 82.2 N/mm^2^, 80.26 N/mm^2^, and 73.76 N/mm^2^. For VE, the VHN was 286.3 N/mm^2^, 282 N/mm^2^, 266.47 N/mm^2^. The highest hardness was found for VM and had values of 680.55 N/mm^2^, 679.93 N/mm^2^, and 558.66 N/mm^2^. In another study by Al-Haj Husain et al. (2022), six additive and six conventional composites were compared. Overall, they found that the additive-manufactured composite materials had lower Vickers hardness than conventional composites. The 3D-printed inkjet polyprint composite material MED690 (ST) had the lowest value among the tested materials. Al-Haj Husain et al. (2022) noted significant differences in the average microhardness values among the materials.

Additionally, Bora et al. (2023) found a significant positive linear relationship between the amount of filler particles and the hardness of a material (*p* = 0.018). Ceramic Crown, which was the only material for permanent restorations researched, had the highest filler weight percentage, higher E-modulus, and hardness among the four additive-manufactured materials [16].

#### 3.1.2. Flexural Strength

**Figure 3:**
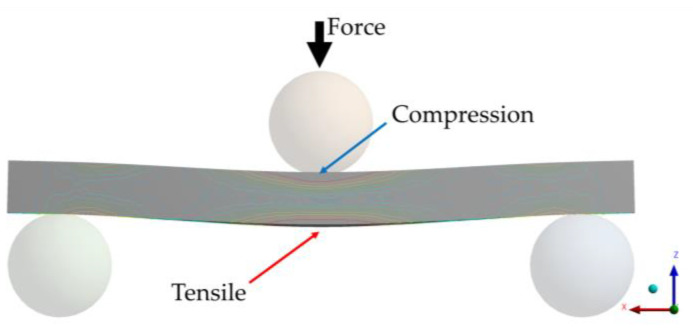
Schematic illustration of the three-point bending test.

Flexural strength, also known as bending strength, refers to the maximum stress that a material can withstand before it breaks when subjected to bending. The included studies measured flexural strength by using the three-point bending test, as shown in Figure 3 by Farhan et al. (2020) [27]. This test also allows for the calculation of the modulus of elasticity, or E-modulus [16]. The threshold required flexural strength for resin materials is 100 MPa (ISO 4049:2019 [28]).

In the study conducted by Bora et al. (2023), conventional composites as well as ceramic materials were compared with additive composite materials. The study found that 3D-printed composites had lower flexural strength (FS) than conventional composites, ceramics, and CAD/CAM-milled composites. Among the four additive composite materials examined, OnX (e-modulus: 5.1 GPa, FS: 131.0 MPa) and Ceramic Crown (e-modulus: 5.9 GPa, FS: 117.4 MPa) exhibited the highest modulus and strength. However, only Ceramic Crown is intended for the fabrication of definitive indirect restorations. Out of the four additive composite materials, C&B MFH and OnX Tough did not reach the threshold of 100 MPa. OnX Tough had a FS of 78.0 MPa and C&B MFH had a FS of 97.1 MPa. Regarding the filler content and flexural strength, no significant difference was observed (*p* = 0.056) [16].

In contrast to the previous research which analyzed differences in flexural strength among various materials, Borella et al. (2023) focused on 3D-printed composites from different manufacturers. They discovered significant variations in flexural strength between the materials studied. Notably, the NNL composite had the lowest value (50 μm: 114.7 MPa, 100 μm: 87.1 MPa), while VSC (50 μm: 124.0 MPa, 100 μm: 115.2 MPa), C&B MFH (50 μm: 125.1 MPa, 100 μm: 118.1 MPa), and RSL (50 μm: 124.2 MPa, 100 μm: 113.6 MPa) had similar values. All the tested materials had values above 100 MPa, except for NNL with a layer thickness of 100 μm. Results showed that the flexural strength of the printed objects was affected by the layer thickness. The VSC, NNL, and RSL groups exhibited significantly lower flexural strength when printed with a layer thickness of 100 μm compared to 50 μm (VSC: *p* = 0.020, NNL: *p* < 0.001, and RSL: *p* = 0.005). Only within the C&B MFH group, no effect was observed on the flexural strength within the two measured layer thicknesses of 50 μm and 100 μm [7].

#### 3.1.3. Elastic Modulus

The elastic modulus (E-modulus), also known as Young’s modulus, is a property to measure a material’s resistance to elastic deformation. It is expressed in pascals (Pa), megapascals (MPa) or gigapascals (GPa) [7]. The E-modulus is an indicator of a material’s stiffness, indicating the ratio between the stress applied to a material and the resulting strain or elastic deformation. This is also known as the proportionality constant between stress and strain, indicating how much stress a material can withstand before it starts to deform [22].

According to Borella et al. (2023), there were variations in the elastic modulus of different 3D-printed composites. The study revealed that the NNL (7.09 GPa) group had the highest elastic modulus, followed by VSC (4.03 GPa), C&B MFH (2.37 GPa), and RSL (1.9 GPa). Furthermore, no significant difference (*p* = 0.970) was found in the E-modulus between materials printed with different layer thicknesses [7]. In contrast, an article by Farkas et al. (2023) investigated the NextDent C&B MFH material and discovered that the E-modulus was highest for specimens printed with a layer thickness of 50 μm and a print orientation of 45°. The value in this case was 3225.4 MPa [21].

A statistically significant relationship was found between the number of filler particles in a composite material and the elastic modulus (*p* = 0.013) [16].

#### 3.1.4. Compression Strength

Compression strength is the maximum amount of compressive load a material can handle before it fails. It is expressed in megapascals (MPa) [21].

The compression tests conducted in the study by Farkas et al. (2023) investigated the compression behavior of NextDent Crown & Bridge MFH (C&B MFH). It was observed that initially, only a small deformation occurred in the material when pressure was applied. The material exhibited elastic properties. In the areas where the pressure was applied, stress is generated. The stress increases linearly with the strain occurring on the contralateral side during the elastic phase. The elastic phase ends when the material enters the plastic phase, after which it enters the yielding domain. From this point, an increase in stress and strain is observed, and once the maximum stress is reached, the material fails. According to Farkas et al., the average maximum compression yield stress value obtained was 85.9 MPa for specimens printed at 0°, while the value was 98.45 MPa for the 45° specimens and around 110 MPa for the 90° specimens. The peak values obtained were 146.64 MPa for 0°, 228.28 MPa for 45°, and 238.26 for 90° [21].

#### 3.1.5. Tensile Strength

Farkas et al. (2023) recorded the highest tensile strength for NextDent C&B MFH specimens printed with a layer thickness of 50 μm. The specimens in this study were also printed at the 50 and 100 μm layer thickness. Tensile strength is the maximum stress that a material can endure before failing under tensile loading and is expressed in megapascals (MPa). The highest stress values were observed for specimens printed with a layer thickness of 50 μm, whereas the highest strain value was observed in specimens printed with a layer thickness of 100 μm. The maximum breaking stress for specimens printed with a layer thickness of 50 μm was around 58 MPa for both print orientations of 0° and 90°, while for the print angle of 45°, a value of 53.7 MPa was measured. Specimens printed with a layer thickness of 100 μm had a maximum breaking stress of 56.8 MPa at a print angle of 0°, and 51.5 MPa and 49.6 MPa at print angles of 45° and 90°, respectively [21].

#### 3.1.6. Fracture Resistance

Fracture resistance is the ability of a material to withstand breakage or cracking under load. It is measured in newtons (N) and indicates the force required to break a material [26]. It is usually used for complex structures such as crowns and bridges when it is not possible to calculate the strength.

Corbani et al. (2021) aimed to investigate the fracture of three-unit fixed dental prostheses made of different materials. The materials included two subtractive materials, fiber-reinforced composite (Trilor, Bioloren) (FRC), high-density polymer (Ambarino, Creamed) (HDP), and one 3D-printed composite, Irix Max (3DP). The findings of the study suggest that there was no significant difference in fracture resistance between 3DP (1360.20 N) and HDP (1312.27 N) (*p* = 0.665). However, a significant difference was observed between 3DP and FRC (839.07 N), with 3DP showing higher fracture resistance than FRC (*p* < 0.001). It was also observed that when fractures occurred in the HDP and 3DP groups, they were mostly repairable failures. Out of the 15 specimens in the HDP group, 11 were repairable fractures, and 4 were non-repairable fractures. The 3DP group (n = 15) exhibited 12 repairable and 3 non-repairable fractures. A repairable fracture was defined as a fracture that remained within the material without extending to the abutment. Most fractures occurring in the HDP and 3DP groups were in the connector area [19].

The article by Suksuphan et al. (2023) compared the fracture resistance of 3D-printed dental crowns (VSC) with milled crowns (Cerasmart (CS) and Vita Enamic (VE)). The study showed that VSC crowns had the lowest fracture resistance. Also, a correlation was found between the occlusal thickness and the fracture resistance from VarseoSmile Crown Plus (VSC) crowns. With an increased occlusal thickness, an increased fracture resistance was observed. The maximum loading force for VSC was 1480.3 N for an occlusal thickness of 0.8 mm, 1629.4 N for 1.0 mm, and 1747.2 N for 1.5 mm. It is worth noting that all crowns were able to withstand normal occlusal loading, even at a minimal occlusal thickness of 0.8 mm. While the article by Suksuphan et al. (2023) focused solely on VSC as a 3D-printed material, the study by Türksayar et al. (2023) also included the Crowntec (CT) material. The measured fracture resistance values showed that CT had the lowest fracture resistance (536 N), significantly differing from Brilliant Crios (BC) (616 N) and VE (803 N) (*p* < 0.011). VSC had a non-significant higher fracture resistance (587 N) than CT, whereas VSC showed a significantly lower fracture resistance than VE (*p* < 0.001) [20,25]. Donmez & Okutan (2022) investigated CT compared to BC, VE, and CS in their study. The average fracture resistance, in increasing order, was CS < BC < VE < CT (1274.32 N < 1333.23 N < 1359.25 N < 1413.91 N). CT had the highest average fracture resistance, although the differences were not significant (*p* = 0.209) [20].

Zimmermann et al. (2018) conducted a study on single molar crowns made of Lava Ultimate (LU), CS, and BC compared to Els 3D harz, along with VE and e.max CAD (e.max) as control groups. Els 3D Harz was assessed in three different material thicknesses (0.5 mm, 1.0 mm, and 1.5 mm). The 2018 study by Zimmermann et al. found that all samples of this material survived the fatigue testing, after which the maximum fracture load was measured. As stated in the study, the maximum fracture load varied significantly within these groups. The highest average loading force was recorded for the 3D-printed material at an occlusal thickness of 1.5 mm, measuring 1478.7 N. Although no significant differences were observed between the different composite materials used in their study (LU, CS, BC, and Els 3D), a significant difference was found between the different types of materials, particularly composites and ceramics (VE and e.max). It can be noted that both the used materials and the occlusal thickness had a significant impact on fracture resistance (*p* < 0.05). Like Suksuphan et al. (2023), Zimmermann et al. (2018) also found a relationship between maximum fracture load and occlusal thickness (*p* = 0.003) [14]. 

In 2020, Zimmermann et al. studied the fracture load of three-unit full-contour fixed dental prostheses. The prostheses were manufactured using both subtractive and additive methods. The subtractive-manufactured materials were BC, Telio CAD, e.max, and inCoris TZI C. The latter two are ceramics, while the others are composite materials. The average fracture load measured was lowest for the Els 3D Even Stronger (Els 3D ES) group (928.9 N), significantly differing from the subtractive-manufactured BC group (1494.8 N) [26].

#### 3.1.7. Wear

Wear is the loss of material on a surface that occurs due to abrasion or erosion over time. This type of wear is multifactorial and can be caused by both physiological and pathological factors. It is an essential property that helps in assessing the durability of a material [22].

A study conducted by Guven et al. in 2023 compared the performance of Crowntec (CT), which is an additively manufactured material, with three subtractive-manufactured materials, including G-CAM (GR), and breCAM.monoCOM (PMMA), and Brilliant Crios (BC). The study found that single molar crowns made from CT had higher external surface deviations than those made from PMMA and GR (*p* ≤ 0.038). Additionally, a greater mesiodistal width discrepancy was observed in CT compared to PMMA and BC (*p* = 0.004), but not for GR. The study concluded that CT exhibited significant surface wear, while BC showed greater volume loss in the wear area. The wear was measured by a computer-controlled mastication simulator. The researchers placed a thin foil between the crown and the steel sphere (antagonist) to simulate three-body wear [22].

Also in 2023, another research group (Türksayar et al., 2023) investigated the two-body wear of standardized screw-retained, implant-supported, single crowns that were manufactured using both additive and subtractive methods. The study compared the performance of CT and VarseoSmile Crown Plus (VSC) with BC and Vita Enamic (VE). The researchers found that the CT and VSC materials had a similar volume loss (*p* = 0.998), which was significantly higher than that of BC and VE materials (*p* < 0.001). In addition, BC and VE materials had comparable maximum wear depths (*p* = 0.999), which were significantly lower than those of other materials (*p* < 0.001) [25]. 

### 3.2. Factors Indirectly Influencing Mechanical Properties

Surface roughness (Sa) is the term used to describe the unevenness or irregularities present on the surface of a material [4,7,18]. These irregularities can be caused by different factors such as wear and manufacturing processes [4,7,18]. The measurement of surface roughness is done by calculating the difference in height between the highest peaks and deepest valleys present on the surface. The result is expressed as the average roughness value (Ra) [4,7,18]. 

Cakmak et al. (2023) compared additive-manufactured materials, Crowntec (CT) and VarseoSmile Crown Plus (VSC), with subtractive-manufactured materials, Brilliant Crios (BC), Vita Enamic (VE), and Vita Mark II (VMII). The result for the highest surface roughness was found before polishing the materials (*p* ≤ 0.026). Notably, VSC exhibited significantly higher surface roughness than the other materials (BC: 0.27 μm, VE: 0.64 μm, and VMII: 0.70 μm) (*p* ≤ 0.001), except for CT (*p* = 0.822). Before polishing, VSC had a Ra of 3.58 μm and CT 2.79 μm. After polishing, the surface roughness of VSC decreased to 0.36 μm and CT decreased to 0.71 μm. All the materials tested did not show a significant difference in surface roughness (BC: 0.15 μm, VE: 0.25 μm, and VMII: 0.17 μm) (*p* ≥ 0.166) [18].

Overall, the thermocycling procedure led to an increase in the roughness of materials, although it was not statistically significant according to Bozoğulları & Temizci (2023). All the average surface roughness values of the measured materials were below or equal to the threshold for plaque accumulation, which is 0.20 μm. In the same study, no significant difference was found between the additive-manufactured materials (CT and permanent crown resin) and Cerasmart. However, a significant difference was observed between VE and VM II compared to the other materials (Bozoğulları & Temizci, 2023).

Research conducted by Borella et al. in 2023 studied the impact of layer thickness (50 and 100 μm) on the surface roughness of different materials in 3D printing. They discovered that the layer thickness had a significant influence on the surface roughness of VSC (*p* < 0.001), C&B MFH (*p* < 0.001), and Resilab 3D Temp (RSL) (*p* < 0.001), but not Nanolab 3D (NNL) (*p* = 0.379). At a layer thickness of 100 μm, C&B MFH showed the highest surface roughness, followed by RSL, which was comparable to VSC. The lowest surface roughness was found for NNL. At a layer thickness of 50 μm, VSC, RSL, and NNL had no significant difference in surface roughness [7].

## 4. Discussion

The current study aimed to provide an overview of the existing literature on the mechanical properties of composites used in 3D printing of definitive indirect restorations. Composites are materials composed of organic and inorganic components such as ceramics, glass ceramics, glass, and composite filler particles embedded in a resin matrix [14,15,29]. Although the manufacturer classifies Nextdent C&B MFH as a long-term temporary composite resin, some articles have investigated this material as a composite for permanent crown and bridge work, such as the studies conducted by Al-Haj Husain et al. (2022) and Farkas et al. (2023), where the materials were subjected to an aging process.

New composite materials have been developed for additive manufacturing to achieve improved aesthetics, color stability, high flexural strength, low abrasiveness, intraoral repair capability, and durability. These materials combine the properties of ceramic materials and composites [24]. In general, composites have high flexural strength and a relatively low modulus of elasticity, allowing them to withstand more elastic deformation before failure [29]. According to the present results, it is clear that it is not enough to consider just one mechanical property when assessing a material’s clinical performance. Therefore, multiple mechanical properties must be taken into account to determine its suitability for a particular application. It is important to mention that as 3D printing of composites for permanent restorations is a recent development, there are limited data available on their mechanical properties [23]. To address this knowledge gap, the current study aims to provide an overview of the existing data on the mechanical properties of 3D-printed composites.

To evaluate the production of dental materials, it is crucial to consider the different properties that impact their performance. These properties can be didactically categorized based on whether they are influenced by the processing technology, the printing technology used, and the substance of the material itself.

Hardness is a property of materials that reflects their resistance to indentation, which can be influenced by wear behavior. It is an important factor in determining the durability of dental restorations [12]. In a study conducted by Cakmak et al. (2023), the surface roughness of both additive- and subtractive-manufactured materials was examined. It was found that the additive-manufactured specimens had lower microhardness values compared to the subtractive-manufactured specimens. One possible explanation for the lower microhardness of additive materials is their difference in composition and manufacturing process [30]. The manufacturing process of CAD/CAM-milled blocks is carried out under standardized conditions, which may have a positive impact on microhardness [31]. In a study conducted in 2022 by Al-Haj Husain et al., it was discovered that the group of composite materials manufactured through additive techniques is less hard in comparison with conventional composites. Among the tested materials, MED690 had the lowest hardness value. Interestingly, this specific material was printed using inkjet poly-print technology, which is a different printing method from the commonly used 3D printing techniques (digital light processing, DLP) in the same study.

The DLP printer is considered superior to the SLA printer in terms of cost and printing speed. However, there is no consensus found in the literature on the accuracy of the DLP printer compared to the SLA printer [9]. The present study did not focus on printing techniques; however, it can be observed that most studies used the DLP printing technique.

The articles included in the current study utilized the three-point bending test to determine the flexural strength of the materials. While this method is valid, it might not be the best method for measuring the flexural strength of brittle materials like ceramics, as suggested by Chung et al. in 2004 [32]. As ceramic materials were used as control groups in the included studies, the three-point bending test used might have influenced the outcome for these materials. However, the uniformity of the test method used in the included studies is favorable.

Flexural strength refers to the amount of force a material can withstand before fracturing, and fractures are one of the most common reasons for restorations to fail, according to Borella et al. (2023). It is required for resin materials to have a minimum flexural strength of 100 MPa (Borella et al., 2023). VarseoSmile Crown Plus, as claimed by the manufacturer, has a flexural strength of 116 to 150 MPa (Suksuphan et al., 2023). Research conducted by Goujat et al. (2018) [31] found that Vita Enamic had an average flexural strength of 148.7 MPa, which is not significantly different from that of VarseoSmile Crown Plus. This is in line with what the manufacturer claims. In the study by Bora et al., 2023, Ceramic Crown, as the only composite resin for permanent indirect restorations, is compared with ceramics materials. This study shows that Ceramic Crown exhibits a flexural strength of 117.4 MPa. This flexural strength is close to the flexural strength of IPS Empress CAD LT (127.65 MPa) measured in the study by Vichi et al. [33]. However, when Ceramic Crown is compared to e.max CAD HT (350.88 MPa) from the same study, it is noticeable that Ceramic Crown exhibits a significantly lower flexural strength. In the case of multi-unit constructions, studies [19,26] show that fractures always occur in the connector area. Complementing the literature, this study showed that not all materials can achieve the threshold to be used as definitive restorative materials. Therefore, the material selection and restoration design should be considered when used for clinical application.

The ability of materials to withstand forces is crucial for their functionality in the oral cavity. During mastication, high occlusal loading forces are exerted. According to studies [19,26], the maximum occlusal mastication force in the molar region is 600 N. However, a study from 2020 by Al-Zordk et al. [34] indicates that the maximum mastication force in the molar region can reach 900 N. Individuals with implant-supported restorations tend to exert more mastication force due to the lack of proprioception. Additionally, individuals with bruxism also tend to exert more mastication force, as mentioned in the study by Martín-Ortega et al. (2022) [35]. Since restorations are subject to large mastication forces, the material used must have high flexural strength [32]. The materials used in the included articles generally had a fracture resistance of more than 900 N, except for the materials used in the study by Türksayar et al. (2023). The difference in the results may be due to the presence of a screw hole in the examined crowns, unlike the full-contour implant-supported crowns examined in the study by Donmez & Okutan in 2022. Furthermore, it is essential to note that the geometry and application of the material also play a significant role in how the material behaves under certain conditions. For instance, a material with a larger occlusal thickness would exhibit higher fracture resistance [36]. However, in the case of tissue-conserving preparation, it may not be possible to provide a certain material thickness. In the study by Zimmermann et al. in 2018, it was found that none of the 0.5 mm ceramic crowns survived the fatigue tests, while the 3D-printed CAD/CAM composite materials with the same occlusal thickness did survive. Suksuphan et al. (2023) also indicated that printed composite materials with a minimal occlusal thickness of 0.8 mm can withstand mastication forces. This allows the use of these printed composites to enable minimal invasive treatments [14]. Therefore, the preparation and restoration design should be optimized to withstand the mastication forces in the oral cavity altered to the restorative material, with the aim of minimal invasive preparation.

Studies by Farkas et al. (2023) and Borella et al. (2023) have shown that print orientation affects not only the accuracy but also the strength of the printed products. According to Farkas et al. (2023), printing at a 90° angle provides the highest compressive strength, while 0° and 45° angles result in lower maximum stress values. Printing at a 90° angle also improves the photopolymerization of the printed layers (Farkas et al., 2023). However, Unkovskiy et al. (2018) [37] suggested that printing at a 45° angle leads to better accuracy. The same research (Unkovskiy et al., 2018) indicates that a 90° print orientation does not affect the mechanical properties, which contradicts the findings of Nold et al. (2021) [38]. In their study, Nold et al. (2021) found that print orientation does indeed have a significant impact on material properties. Their conclusion aligns with the current study’s findings. Furthermore, Farkas et al. (2023) also found that the print orientation affects the modulus of elasticity.

The modulus of elasticity is an essential factor in resisting deformation under stress [22]. The stiffer the material, the higher its modulus of elasticity, and the more stress it can withstand without permanent deformation [23]. When it comes to creating indirect restorations, the ideal material should have a modulus of elasticity equal to that of enamel [7], which ranges from 74 GPa to 130 GPa according to different literature [39]. However, the materials that were tested in the research had a modulus of elasticity lower than enamel. This is beneficial for wear on the opposing tooth, as confirmed in the study by Lawson et al. (2016) [40]. However, the lower modulus of elasticity of composite resins makes them more susceptible to debonding compared to rigid materials (Zimmermann et al., 2018). Additionally, the modulus of elasticity of these materials was even lower than that of dentin (7–13 GPa) [24,26], which may lead to stress accumulation in the remaining tooth structure [7].

Zimmermann et al. (2020) conducted a study that shows how CAD/CAM composites are resilient materials that can absorb forces and deflect destructive fracture energy, unlike rigid lithium disilicate glass ceramics. Some 3D-printed composites have a lower strength and modulus of elasticity, but they can absorb more energy, making them more suitable for specific applications. For instance, composites with a low modulus of elasticity are useful for applications where flexibility is acceptable, such as temporary full-arch prostheses or removable appliances. On the other hand, composites with a higher modulus of elasticity, such as Ceramic Crown, are suitable for making definitive crown and bridge work that must withstand high occlusal loads and should not bend [16]. The modulus of elasticity of the restorative material is crucial, but so is that of the underlying structure [41]. For implant-supported restorations, a material with a low modulus of elasticity, such as composite, is advantageous for absorbing forces, given the absence of a periodontal ligament [25]. 

In dentistry, restoration material needs to be wear-resistant, which means that the material should not lose much of its surface due to wear [42]. It is ideal for dental materials to have Vickers hardness similar to enamel, which ranges from 320 to 380 kg/mm^2^ [43]. Generally, harder materials are considered to be more wear-resistant [7]. Some 3D-printed composites are found to be more wear-resistant than the harder lithium disilicate material, as per previous research [40], contrary to expectations. However, the current study did not compare the wear between printed composites and lithium disilicate ceramic. In a study conducted by Guven et al. in 2023, it was found that the 3D printing material Crowntec experienced more wear on both its external surface and mesiodistal width compared to subtractive-manufactured crowns. This could be due to the fabrication procedure, as additive-manufactured crowns require additional polymerization [10]. Wear can lead to dimensional changes, causing interproximal contact loss, reduced occlusion, and altered crown margins over time [22]. An alternative explanation for higher wear in composites is the adverse effect of water absorption. After absorbing water, composites become softer or undergo hydrolysis of the silane coupling agent [44]. 

Another crucial aspect is the margin adaptation that can affect the clinical success of the printed restoration. It refers to the distance between the preparation outline and the margin of the restoration [20]. Favorable margin adaptation results in minimal cement thickness, reducing the risk of prosthetic failure [45]. The studies by Donmez & Okutan (2022) and Suksuphan et al. (2023) show that additive-manufactured indirect restorations have better margin adaptation than subtractive-manufactured restorations.

Polishing printed materials can enhance their wear resistance by reducing surface roughness. Moreover, polishing can also eliminate non-polymerized monomers from the surface after the post-polymerization process. In a study conducted by Türksayar et al. (2023), all tested materials displayed higher maximum wear than 17.3 μm, which is equivalent to the physiological annual wear of enamel in the pre-molar region. However, the study of Türksayar et al. (2023) did not involve polishing or glazing the materials, which resulted in increased surface roughness. Surface roughness is one of the factors that indirectly affects material properties.

In terms of finishing procedure, restorations can be polished or left unpolished, which impacts their surface roughness. Increased surface roughness can lead to bacterial adhesion [46], wear to the antagonist, and discoloration [31,47]. Therefore, restoration materials need to have a surface roughness below the threshold value of 0.2 μm, which is considered clinically acceptable [18,23]. Unpolished materials often result in a matte finish and less aesthetic appearance [23]. Within the included articles in the current study, the study by Cakmak et al. (2023) found that none of the tested materials had a surface roughness below the threshold value before polishing. Polishing reduced the surface roughness of all materials, with some showing a significant difference and others not. According to Cakmak et al. (2023), VarseoSmile Crown Plus and Crowntec were the roughest before polishing. However, after polishing, there was no statistically significant difference compared to the other materials. Two of the tested materials (Brilliant Crios and Vita Mark II) achieved a reduction in surface roughness to below the threshold value after polishing. In contrast, the study by Bozoğulları & Temizci (2023) found that all tested materials showed a surface roughness below the threshold value after polishing. This difference could be due to the polishing method used in each study. Bozoğulları & Temizci (2023) sanded the specimens with 600-, 1000-, and 1200-grit sandpaper before polishing with medium to superfine (SofLEX, 3M) polishing discs, while Cakmak et al. (2023) used the manufacturer’s recommended polishing method for each material.

In addition to surface roughness, the filler percentage also influences the mechanical properties of printed materials [7]. Changing the proportion and type of filler particles in the composite can improve the elasticity modulus and properties such as tensile strength, hardness, and wear resistance [23]. However, the effect of filler percentage on flexural strength is still up for debate. Bora et al. (2023) found that the filler percentage has no impact on flexural strength, contrary to research by Zimmermann et al. (2020). Additionally, filler particles themselves can improve flexural strength by deflecting cracks, thereby stopping it to some extent and preventing the material from collapsing immediately [26]. This difference in results could be due to the composition of the composite material. Ceramic filler particles have been added to 3D resin composite to create permanent restorations from 3D-printed composites [7]. For permanent fabrication of indirect restorations, resins should have a minimum filler percentage of 50% [16]. While adding filler particles can have a positive effect on mechanical properties, it is important to note that it can also affect the fluidity of the printing fluid as well as the complexity of printing process [4]. 

According to the current study, VarseoSmile Crown Plus can withstand a lower maximum load force compared to subtractive-manufactured materials. This may be due to the lower flexural strength and fewer filler particles. However, an advantage of this composition is the fluidity of the material during the printing process. To increase the maximum load force of VarseoSmile Crown Plus while maintaining its fluidity during printing, the occlusal thickness of the restorations may need to be increased to more than 1.0 mm [24].

It can be said that the mechanical properties of 3D-printed materials are influenced by various printing parameters, such as printer technology, print orientation, layer thickness, and post-processing techniques [21]. Post-processing involves a series of steps, including removing the printed object from the print platform, cleaning, post-polymerization of the unreacted monomers, and removing supports [48]. By altering these parameters, it is possible to manipulate the material properties to make the final printed product more suitable for its intended clinical application [21]. 

When it comes to 3D printing, adjusting the thickness of each layer affects the time it takes to complete the print. However, doubling the layer thickness does not mean the printing time is halved. This is because thicker layers require more time to harden. Nonetheless, a study by Borella et al. (2023) found that printing with a layer thickness of 100 μm can reduce printing time by 40%. However, the same study found that objects printed with a layer thickness of 100 μm did not produce the best results when compared to those printed with a layer thickness of 50 μm. This finding is supported by previous research from Zhang et al. (2019) [9], which concluded that DLP and SLA printers produce more accurate objects when using a layer thickness of 50 μm rather than 100 μm. Additionally, if a 100 μm layer thickness is chosen, the DLP printer is more accurate than the SLA printer, according to Zhang et al. (2019).

In the field of dentistry, 3D-printable materials must be able to withstand the harsh conditions present in the mouth, which can greatly affect the long-term performance of restorations [12,21]. These conditions include humidity, temperature changes, food or beverage intake, and oral hygiene. Therefore, it is essential to conduct thorough testing of materials before they are brought to the market for clinical applications in patients. Although it is not possible to examine all specific material properties in vitro, in vitro research provides a good indication of what can be expected in the mouth [23]. For example, thermocycling and water storage are commonly used artificial aging techniques to simulate the conditions found in the mouth [12].

The survival of dental restorations is impacted not only by patient-related factors such as compliance but also by the practitioner [4,12,18]. It is crucial to strictly adhere to the manufacturer’s prescribed procedures, including correct processing procedures and cementation steps [14]. Neglecting these can have a significant impact on the long-term survival of dental restorations [4,12,18].

Although ceramic materials are still considered aesthetically superior, composites have several advantages. Firstly, they are easier to repair intraorally [14,19,20]. Secondly, they can better absorb forces as their modulus of elasticity matches that of dentin better than ceramic materials. These shock-absorbing properties make them a good solution for implant-supported restorations [20]. Moreover, additive composite materials may be suitable for the fabrication of a restoration on a minimally invasive prepared tooth structure [14,24]. This kind of preparation is clinically preferable as it preserves tooth tissue [49], reduces post-operative pain or sensitivity, and increases the chance of maintaining the vitality of the respective tooth. Results from Zimmermann et al. (2018) have shown that composite restorations with a thickness of 0.5 mm survive fatigue tests and exhibit a fracture resistance greater than the expected posterior occlusal load. Despite manufacturers not recommending their materials below a minimum thickness, these thin restorations offer an alternative treatment to avoid pulp exposure in patients with limited occlusal space or in young children [24]. In addition, it is known that composites are less brittle compared to ceramics and have better damage tolerance. This feature results in smoother margins and less chipping at the edge [19]. Additionally, it can be said that the additive manufacturing process is cost-effective, both in terms of production and the required equipment investment [45].

It is important to acknowledge certain limitations in the current study when interpreting the results. One of the main issues is the use of various measurement methods to measure a specific mechanical property. As a result, each measurement method can give different outcomes when measuring the same material. Besides the measurement method, the geometry of the specimens is also a variable that varied in the included articles. This variation may have influenced the outcome of the study, making it difficult to compare studies with different geometries. The included studies used different samples such as bars, printed crowns, implant-supported restorations, and bridges. Additionally, various factors in the mouth can affect the mechanical properties of a dental material. These factors include enzymes and proteins in saliva, parafunction, such as mouth breathing, and foreign objects like piercings. These factors were not considered in the studies mentioned in the current research. In addition, the studies used different printing technologies and parameters. Some studies employed DLP printers, while others used SLA or Polyjet printers. Moreover, there was variation in post-processing, print orientation, and layer thickness between the studies. In the current research, various mechanical properties were investigated, but not all of them were evaluated under similar conditions. Additionally, other mechanical properties are relevant to dentistry but were not explicitly examined, such as ductility, malleability, Poisson’s ratio, shear strength, and torsional strength. Also, fracture toughness is an important mechanical property that needs to be researched. Fracture toughness describes the material’s resistance against fracture propagation. Overall, it can be concluded that the results cannot be generalized, even for the same materials.

**Figure 4:**
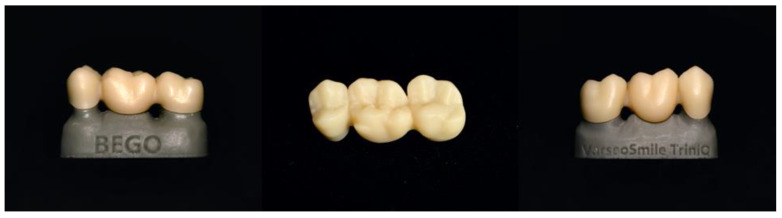
Fixed dental prosthesis printed with VarseoSmile TriniQ, BEGO.

As 3D printing in dentistry is a relatively new technology, there is limited scientific research available on printing definitive composite restorations. While some mechanical properties of the composite resins are summarized in the current research, there is still much research to be performed on both the mechanical properties of the composite resins and recently developed ones, such as VarseoSmile TriniQ, BEGO (Figure 4). Future research could focus on the adhesion of 3D-printed indirect composite restorations for long-term survival, along with various printing parameters that influence material properties, such as layer thickness and print orientation. Additionally, it may be valuable to investigate different printing technologies, such as inkjet Polyjet printers, which may be useful for the esthetic zone. It is important to conduct research that compares 3D-printed indirect restorations with each other, with subtractive-manufactured restorations, and with conventional composites. This should be examined not only in vitro but also in vivo.

Recently, a clinical trial investigated the clinical survival behavior (up to one year) of posterior fixed dental prostheses made from 3D-printed resin composite material (ELS Even Stronger, Saremco, Switzerland). The researchers reported that the tested material was found to be acceptable for long-term provisional use after a one-year follow-up. However, they emphasized the need for further follow-up to determine the long-term survival and to assess whether these materials can be an alternative for permanent indirect restorations. According to their study, the survival rate after one year was 71.6% (Kaplan–Meier) [50]. This example of a well-developed clinical trial is scarce and considerations about other 3D-printable materials lacking in the literature. As a suggestion, further studies consisting of mouth-split randomized clinical trials [51] should be designed to compare how different 3D-printed restorations behave in the same environment.

In vivo research should examine whether indirect composite restorations maintain their stability in the oral environment and their long-term survival, taking into account different conditions that may occur in the mouth, such as parafunction. Finally, biocompatibility is a critical aspect that should not be overlooked in future research [52].

## 5. Conclusions

The aim of this scoping review was to provide an overview of the literature about the mechanical properties of composites used in 3D printing for definitive restorations. Within the limitations of the current research, the review found:

- Composite materials for 3D-printed dental restorations have lower hardness and flexural strength compared to ceramics and conventional composites. Fracture resistance results are inconclusive, but repairs are often possible. Thickness influences compressive strength, with printed crowns weaker than subtractive-manufactured ones.
- Additive materials start rougher but smooth out after polishing. However, there is no consensus over whether their roughness exceeds plaque accumulation thresholds.
- In summary, 3D-printed composites do not yet meet the proper mechanical properties to function as long-lasting permanent restorations. It is essential to approach the clinical application of these materials with a critical mindset due to the limited amount of available evidence. Currently, there is a need for long-term in vivo studies on the use of additive-manufactured restorations.

Although current 3D-printed composites do not yet meet the required mechanical properties for use as definitive indirect restorations, the authors do see value in their specific clinical application. This includes phased restorative work where the bite needs to be raised and/or for people who, due to dysfunctional grinding/clenching, are often not eligible for other restorative materials.

## Figures and Tables

**Table 1:** Summary of the included articles.

Study	Material and Sample Size	Material Manufacturer	Material Indication	Printer Technology	3D Printing Parameters	Measurements	Geometry of 3D Printed Specimens	Results
Al-Haj Husain et al., 2022 [12]	Additive manufactured: 1. Optiprint Temp (OP) 2. C&B MFH (ND) 3. Crowntec (SA) 4. Temp Print (TP) 5. 3DELTA ETEMP (DM) 6. MED690 (ST) Conventionally manufactured: 7. Gradia Direct (GR, GC) 8. Clearfil Majesty (CM) 9. Tetric EvoCeram (TE) 10. Gradia Direct Flo (GR-F, GC) 11. Clearfil Majesty Flow (CM-F) 12. Tetric EvoFlow (TE-F) A total of *n* = 240 was measured. For each material group *n* = 20, with *n* = 10 for the aged and *n* = 10 for the non-aged materials.	1. Dentona 2. NextDent 3. Saremco Dental AG 4. GC 5. Deltamed 6. Stratasys, Ltd. 7. GC 8. Kuraray Noritake 9. Ivoclar AG 10. GC 11. Kuraray Noritake 12. Ivoclar AG	The additive-manufactured polymers tested in this study were marketed for interim restorations, except for one material (Saremco print CROWNTEC; Saremco Dental AG) which was also marketed for definitive restorations. The conventional composite resins were marketed for definitive restorations.	Specimens of all groups except of ST printed with a DLP 3D printer (Asiga Max UV; Asiga). The group ST was processed with the PolyJet printing technology by using the corresponding printer (Stratasys J700; Stratasys, Ltd.).	The position of the build platform, printing thickness (50 μm), and orientation (round surface facing the build platform) were identical in all groups. The specimens were submerged in an ultrasonic bath with 99% isopropyl alcohol for 5 min. After cleaning, the specimens were retrieved, dried, and polymerized in an ultraviolet (UV)-polymerization machine (Otoflash Post Curing Light Pulsing Unit; EnvisionTEC) with full spectrum (300 to 700 nm) for 10 min.	Hydrothermal aging Vickers hardness	Cylindrical shaped specimens Ø10 × 2 mm	The choice of the material had a significant effect and resulted in lower hardness for the three-dimensionally printed materials than for the conventional composite resins. Under fatigue conditions, the choice of the material showed no significant difference when the Vickers microhardness was evaluated.
Bora et al., 2023 [16]	Additive manufactured: 1. C&B MFH 2. Ceramic crown 3. OnX 4. OnX Tough Reference materials: 5. Lava Ultimate (A milled composite resin) 6. Filtek Supreme (A conventional composite) 7. IPS e.max CAD (A ceramic) A total of *n* = 70 was measured. For each material group *n* = 10.	1. NextDent 2. SprintRay 3. SprintRay 4. SprintRay 5. 3M 6. 3M 7. Ivoclar AG	1. 3D-printed resin for provisional crowns 2. 3D-printed resin for permanent crowns 3 and 4. 3D-printed resin for provisional hybrid dentures 5. Milled resin-composite for permanent crowns 6. Light-cured resin composite for direct fillings 7. Milled ceramic for permanent crowns	All 3D-printed resin materials were printed in a vat digital light processing (DLP) printer (Pro 55, SprintRay, Los Angeles, CA, USA).	The specimens were positioned on the build platform such that the long axis of the bar was oriented parallel to the build platform (parallel with the horizon) and printed with a 100 μm layer thickness. Specimens were cleaned using 91% ethanol and post-cured in a cure box (ProCure II, SprintRay).	Filler weight percentage (wt%) Filler and resin composition Flexural strength/modulus Vickers hardness	Rectangular blocks 2 mm × 2 mm × 25 mm (flexural strength/modulus) Rectangular blocks 4 mm × 4 mm × 6 mm (Hardness)	All 3D-printed resins, for both interim and definitive restorations, had significantly lower flexural strength, modulus, and hardness than the conventional and milled resin composites and ceramic material (*p* < 0.001).
Borella et al., 2023 [7]	Additive manufactured: 1. VarseoSmile Crown Plus (VSC) 2. NextDent C&B MFH (MFH) 3. Nanolab 3D (NNL) 4. Resilab 3D Temp (RSL) A total of *n* = 80 was measured. For each material group *n* = 10 at 50 μm and *n* = 10 at 100 μm.	1. Bego 2. Vertex-Dental B.V. 3. Wilcos do Brasil Ltda 4. Wilcos do Brasil Ltda	1. A ceramic-filled hybrid material for 3D printing of permanent restorations 2. 3D-printed resin for provisional crowns 3. Recommended for printing veneers, onlays, inlays, and bridges 4. Recommended for printing inlays, onlays, crowns, and temporary bridges	Digital light processing (DLP) (Anycubic Photon Mono, Anycubic 3D)	Resins printed at two different layer thicknesses (50 and 100 μm). Build orientation: 45°. The specimens were washed with 99.5% p.a. isopropyl alcohol for 5 min and light-cured for 30 min.	Surface roughness (μm) Flexural strength (MPa) Elastic modulus (GPa) Vickers hardness (N/mm^2^)	Bars 25 mm × 2 mm × 2 mm	VSC showed the highest VHN for 50 μm (27.6 N/mm^2^). From the four tested resins, VSC had the second highest E-modulus in both layer thicknesses (50 μm: 4.51 GPa and 100 μm: 4.03 GPa). The flexural strength (FS) for VSC differs within the layer thicknesses. For 50 μm, an FS of 124 MPa was found, whereas 100 μm had an FS of 115.2 MPa. The surface roughness of VSC also differs within the layer thicknesses. The highest value was found for 100 μm (50 μm: 0.23 and 100 μm: 0.27 μm). The layer thickness affected all performed tests except the elastic modulus.
Bozoğulları & Temizci, 2023 [4]	Additive manufactured: 1. Crowntec (SC) 2. Permanent Crown Resin (FP) Subtractive manufactured: 3. Cerasmart 270 (CS) 4. Vita Enamic (VE) Reference material: 5. Vita Mark II (feldspathic glass ceramic) (VM) A total of *n* = 150 was measured. For each material *n* = 10 for surface roughness and *n* = 20 for color stability and stainability.	1. Saremco Dental AG 2. Formlabs Inc. 3. GC Corp. 4. Vita Zahnfabrik 5. Vita Zahnfabrik	For definitive restorations	1. DLP (Asiga MAX U, Asiga, Sydney, Australia) 2. SLA (Form 3, Formlabs, Somerville, MA, USA)	All specimens were printed with a layer thickness of 50 μm and a build orientation of 90°. The DLP specimens were cleaned with an alcohol-soaked (96%) cloth and then subjected to a post-polymerization process with 4000 lighting exposures using a polymerization device. The SLA printed specimens were washed with 99% isopropyl alcohol for 3 min using ultrasonic cleaning to remove any excess resin; the dried specimens were then subjected to a post-polymerization process using FormCure (Formlabs, Somerville, MA, USA) for 30 min at 60 °C according to the manufacturer’s recommendations.	Color stability Stainability Surface roughness (Ra) (The Ra values and the color parameters were measured before and after thermocycling)	Rectangular blocks 14 mm × 12 mm × 2 mm	Based on the data of the study, tested 3D-printed permanent composite resins showed similar or lower roughness values than tested milled CAD/CAM materials, which were clinically acceptable values. All investigated materials showed acceptable surface roughness after thermocycling that was equal to or below the plaque accumulation threshold of 0.2 μm. The thermocycling increased the roughness of all tested materials. However, this increase was not significant.
Cakmak et al., 2023 [18]	Additive manufactured: 1. Crowntec (CT) 2. VarseoSmile Crown Plus (VS) Subtractive manufactured: 3. Brilliant Crios (BC) (a reinforced composite) 4. Vita Enamic (VE) (a polymer-infiltrated ceramic network) 5. Vita Mark II (VM) (a feldspathic ceramic) A total of *n* = 50 was measured. For each material *n* = 10.	1. Saremco Dental AG 2. Bego 3. Coltène AG 4. Vita Zahnfabrik 5. Vita Zahnfabrik	For definitive restorations	A digital light processing (DLP) printer (MAX UV; Asiga).	Specimens were printed with a 50 μm layer thickness. After fabrication, CT specimens were cleaned with an alcohol-soaked (96%) cloth until all resin residues were completely removed, while vs. specimens were ultrasonically cleaned in ethanol for 5 min (3 min of pre-cleaning in reusable ethanol and an additional 2 min in fresh ethanol). Specimens were then air-dried and light-polymerized either with 4000 (CT, 2 × 2000) or 3000 (VS, 2 × 1500) light exposures (Otoflash G171; NK Optik) under a nitrogen oxide gas atmosphere.	Surface roughness Optical properties Vickers microhardness (Measuring took place before and after stimulated brushing and coffee thermal cycling)	Disk-shaped specimens Ø10 × 1 mm	Tested additive-manufactured resins can be considered more susceptible to simulated brushing and coffee thermal cycling than the other materials, given the fact that their surface roughness and color difference values were higher than previously reported acceptability thresholds and because they had the lowest microhardness after all procedures were complete.
Corbani et al., 2021 [19]	Additive manufactured: 1. Irix Max (3DP) (nanocomposite) 2. Starbond CoS powder 30/Super Porcelain EX3 (the metal-ceramic group) (MC) Subtractive manufactured: 3. Trilor (Fiber-reinforced composite, layered with a nanocomposite (G-aenial Sculpt) (FRC) 4. Ambarino (monolithic high-density polymer) (HDP) A total of *n* = 60 was measured. For each material *n* = 15.	1. DWS 2. Scheftner.dental 3. Bioloren 4. Creamed	For definitive restorations	1. SLA-based 3D printer (DFAB, DWS, Thiene, Italy)	The thickness of build layer was 50 μm and the maximum laser speed was 5000 mm/seconds. The printed specimens were cleaned with 95% ethanol for 1 min and post-cured using ultraviolet curing unit (Dcure, DWS) for 6 min as per the manufacturer’s instructions.	Fracture resistance	Three-unit fixed dental prosthesis (FDP) to replace a missing second maxillary pre-molar	Three-unit FPDs made with monolithic materials, such as 3DP composite and HDPs, showed better fracture resistance in comparison to the layered CAD/CAM FRC FPDs, where chipping was the most common type of failure; 3D-printed and milled composite-based materials might offer a suitable solution for the fabrication of FDPs.
Donmez & Okutan, 2022 [20]	Additive manufactured: 1. Crowntec (SP) Subtractive manufactured: 2. Brilliant Crios (BC) 3. Vita Enamic (VE) 4. Cerasmart 270 (CS) A total of *n* = 40 was measured. For each material *n* = 10.	1. Saremco Dental AG 2. Coltène AG 3. Vita Zahnfabrik 4. GC Corporation	For definitive restorations	Digital light processing-based 3D printer (MAX UV; ASIGA, Sydney, Australia)	Parameters were set to a thickness of 50 μm, exposure time of 1.8 s, maximum light intensity of 12.14 mW/cm2, z compensation of 0 μm, and xy compensation of 0 μm. Following the printing process, the external surfaces of the crowns were cleaned with an alcohol-soaked (96%) cloth, while the internal surfaces were cleaned with a brush soaked in an alcohol solution until all resin residues were completely removed. Then, crowns were dried by using an air syringe and were light cured with 4000 lighting exposures by using a Xenon lamp-curing device (Otoflash G171; NK Optik, Baierbrunn, Germany) under nitrogen oxide gas atmosphere.	Marginal gap Fracture resistance (maximum load to failure in N)	Full contour maxillary first pre-molar crown. The height of the crown was 9 mm from the buccal aspect and 8.5 mm from the palatal aspect, while the thickness of the restoration was 2 mm in the proximal surfaces, 2.5 mm in the buccal and palatal surfaces, and the minimum occlusal thickness was 1.5 mm. The cement gap used was 25 μm, while the extra cement gap (cement spacer) was set to 50 μm.	Material type did not affect fracture resistance values (*p* = 0.209). Implant-supported 3D-printed composite crowns showed higher marginal adaptation compared with the milled crowns before and after cementation. In addition, all crowns endured similar forces before fracture.
Farkas et al., 2023 [21]	NextDent C&B Micro-Filled Hybrid (MFH) A total of *n* = 36 specimens was measured. With *n* = 24 for tensile strength testing and *n* = 12 for compression testing.	3D Systems	To produce long-term temporaries, crown, and bridges	DLP (Digital light processing) “ANYCUBIC Photon Mono X” 3D printer	Printing at different layer angulations 0°, 45°, and 90°. Different layer thicknesses 100 μm and 50 μm. After printing, all specimens were rinsed multiple times in an alcohol solution (96%) and treated with UV light according to the specifications given by the resin manufacturer.	The influence of printing layer direction and thickness on the tensile and compression properties.	Tensile and compression test specimens were modeled at 0°, 45°, and 90°.	The highest tensile values were obtained for specimens printed with a layer thickness of 0.05 mm. In conclusion, both printing layer direction and thickness influence mechanical proprieties and can be used to alter the materials’ characteristics and make the final printed product more suitable for its intended purposes.
Guven et al., 2023 [22]	Additive manufactured: 1. Crowntec (MS) Subtractive manufactured: 2. G-CAM (GR) 3. breCAM.monoCOM (PMMA) 4. Brilliant Crios (BC) A total of *n* = 40 was measured. For each material *n* = 10.	1. Saremco Dental AG 2. Graphenano Dental Sl 3. Bredent GmbH) 4. Coltène AG	For definitive restorations	A digital light processing 3-dimensional printer (MAX UV; ASIGA, Sydney, Australia)	MS crowns with 50 μm layer thickness	External surface Mesiodistal width Wear on the occlusal surfaces	A complete mandibular right first molar crown that had 30 μm cement gap, 3 mm thick axial walls, 1.5 mm thick margins, and 1 mm of minimum occlusal thickness.	The additive-manufactured composite resin was more prone to deviations, while reinforced composite resin had lower wear resistance than most of the tested materials.
Sandmair et al., 2023 [23]	VarseoSmile Crown Plus (A ceramic filled hybrid material for 3D printing) A total of *n* = 3 was measured.	Bego	For definitive restorations	Digital light processing printer (DLP).	The printing layers were perpendicular to the direction of loading. Post-processing followed the manufacturer’s instructions and consisted of an ultrasonic bath in isopropanol, followed by light curing in the Otoflash (BEGO, Bremen, Germany) with two cycles of 1500 flashes each.	Surface roughness measured through the three-point flexure test.	Rectangular blocks 80 mm × 10 mm × 4 mm	Before and after flexure testing was performed; a higher roughness (Sa) was found for VSC than for conventional materials. However, the Sa for VSC is still clinically acceptable. Furthermore, this study showed that AFM surface analysis is a suitable procedure to investigate surface changes in 3D-printed dental materials.
Suksuphan et al., 2023 [24]	Additive manufactured: 1. Varseosmile (VS) Subtractive manufactured: 2. Cerasmart (CE) (hybrid nanoceramic) 3. Vita Enamic (VE) (polymer-infiltrated ceramic network (PICN)) A total of *n* = 90 was measured. For each material (*n* = 30) three different occlusal thicknesses were made *n* = 10 for 0.8 mm, *n* = 10 for 1.0 mm and *n* = 10 for 1.5 mm.	1. Bego 2. GC Corporation 3. Vita Zahnfabrik	For definitive restorations	DLP based printer, FreeForm Pro 2 (ASIGA; Anaheim Hills, CA, USA)	Post-cured to the manufacturer’s recommendations. All crowns were cleaned with ultrasonic baths, air-dried.	Marginal adaptation Fracture resistance	A mandibular first molar based on the preparation guidelines for full ceramic crowns (1.0 mm occlusal reduction, 1.2 mm proximal, axial reduction with 6 degrees of convergence profile, 0.8 mm deep chamfer crown margin, and round line angles). Cement spacer was set to 50 μm. Single-molar crowns with occlusal thicknesses of 0.8, 1, and 1.5 mm.	For all occlusal thicknesses, the vs. crowns demonstrated the lowest AMD and MG distances, significantly different from those of the other two milling groups (*p* < 0.05), whereas CE and VE did not differ significantly (*p* > 0.05). All vs. crowns were fractured using the lowest loading forces (1480.3 ± 226.1 to 1747.2 ± 108.7 N). No CE and 1 and 1.5 mm VE crowns fractured under a 2000 N maximum load. All hybrid-material crowns demonstrated favorable marginal adaptation within a clinically acceptable range, with 3D printing yielding superior results to milling. All materials could withstand normal occlusal force even with a 0.8 mm occlusal thickness.
Türksayar et al., 2023 [25]	Additive manufactured: 1. Crowntec (CT) 2. VarseoSmile Crown Plus (VS) Subtractive manufactured: 3. Brilliant Crios (BC) (reinforced composite resin) 4. Vita Enamic (EN) (polymer-infiltrated ceramic network) A total of *n* = 40 was measured. For each material *n* = 10.	1. Saremco Dental AG 2. Bego 3. Coltène AG 4. Vita Zahnfabrik	For fixed definitive prostheses	A digital light processing-based 3-dimensional (3D) printer (MAX UV; ASIGA)	Occlusal surface facing the building platform. A 50 μm layer thickness. After fabrication, residual resin on the CT crowns was cleaned with 96% alcohol-soaked cloth, whereas vs. crowns were cleaned in an ultrasonic bath containing 96% ethanol solution for a total of 5 min (3 min of pre-cleaning in reusable ethanol and 2 min of cleaning in fresh ethanol). After cleaning, all crowns were dried with an air syringe. CT crowns were then placed in a xenon polymerization device (Otoflash G171; NK Optik) and polymerized under nitrogen oxide gas atmosphere (2000 × 2 lighting exposures). Thereafter, vs. crowns were placed in the same xenon polymerization device and polymerized under nitrogen oxide gas atmosphere (1500 × 2 lighting exposures).	Volume loss (mm^3^), maximum wear depth (mm^2^), and fracture resistance after thermomechanical aging	Standardized screw-retained, implant-supported maxillary right first pre-molar crowns, with screw access channel.	CT and vs. had higher volume loss and maximum wear depth than BC and EN (*p* = 0.001). Additive-manufactured screw-retained, implant-supported crowns had higher volume loss and maximum wear depth.
Zimmermann et al., 2018 [14]	Additive manufactured: 1. els-3D (3D-printed composite) (3D) Subtractive manufactured (particle filled composites): 2. Lava Ultimate (LU) 3. Cerasmart (CE) 4. Brilliant Crios (BC) Control groups: 5. Vita Enamic, hybrid ceramic (VE) 6. e.max CAD, lithium disilicate ceramic (EC) A total of *n* = 180 was measured. For each material (*n* = 30), three different occlusal thicknesses were made *n* = 10 for 0.5 mm, *n* = 10 for 1.0 mm and *n* = 10 for 1.5 mm.	1. Saremco Dental AG 2. 3M ESPE 3. GC Corporation 4. Coltène AG 5. Vita Zahnfabrik 6. Ivoclar Vivadent AG	For definitive restorations	DLP 3D printing device (Freeform Pro 2, ASIGA; Anaheim Hills, CA, USA)	The spacer parameter was set to 80 μm. Parameters were set to slice thickness 50 μm, exposure time 0.6 s, minimum/maximum light intensity 13.14 mW/cm2, z compensation 0 μm, xy compensation 0 μm. Crowns of group 3D were first cleaned and washed in isopropanol 98% for 2 × 3 min using ultrasonic and then light cured with 4000 lighting exposures using Otoflash G171 device under nitrogen oxide gas atmosphere.	Fatigue Fracture load	Single molar crown	All particle-filled composite crowns with 0.5 mm thickness survived fatigue testing. The maximum fracture loading forces significantly varied among the groups tested. There was a statistically significant two-way interaction between material and thickness, *p* = 0.003.
Zimmermann et al., 2020 [26]	Additive manufactured: 1. els 3D resin even stronger (3D) Subtractive manufactured: 2. Brilliant Crios (BC) 3. Telio CAD (TC) 4. e.max CAD (EX) 5. inCoris TZI C (TZ) A total of *n* = 60 was measured. For each material *n* = 12.	1. Saremco Dental AG 2. Coltène AG 3. Ivoclar Vivadent AG 4. Ivoclar Vivadent AG 5. Dentsply Sirona	Manufacturers’ recommendation for indications of use for the respective CAD/CAM materials is up to a permanent four-unit FDP for group TZ, up to a permanent three-unit FDP up to the second bicuspid as distal abutment for group EX, and up to a four-unit temporary FDP for group TC. Indications of use for groups BC and 3D are limited to permanent single-tooth restorations.	The Asiga Freeform PRO2 DLP printer (ASIGA; Anaheim Hills, CA, USA)	Thickness at 50 μm, exposure time at 1 s, minimum/maximum light intensity at 18.34 mW/cm2, z compensation at 0 μm, and xy compensation at 0 μm. Post-processing protocol for FDPs of group 3D comprised cleaning and washing in isopropanol for 2 × 5 min using ultrasonic cleaner with a subsequent light curing with 4000 lighting exposure using a Xenon lamp curing device with a N2-gas atmosphere (Otoflash G171; NK Optik, Baierbrunn, Germany) (2 flashlight lamps, wavelength range 280–580 nm, peaks at approximately 480 and 530 nm).	Fracture load (N)	Three-unit full contour fixed dental prostheses (FDP’s) replacing one molar, 36. Bridge 35–37 connector size 16 mm^2^; anatomic-ovoid-shaped connector design; minimum occlusal thickness 1.5 mm.	Statistically significant differences were found for the fracture load of three-unit FDPs fabricated from different CAD/CAM materials (*p* < 0.05). The highest mean fracture load was found for group TZ (2099.5 ± 382.1 N). Group 3D showed the lowest mean fracture load (928.9 ± 193.8 N). Group BC performed statistically significantly differently from group 3D with a mean fracture load of 1494.8 ± 214.5 N (*p* < 0.05). Particle-filled composite resin CAD/CAM materials showed fracture load values within the range of ceramic materials with a specific indication of use for three-unit FDPs.

## Data Availability

Data are contained within the article.

## Supplementary Materials

The following supporting information can be downloaded at: https://www.mdpi.com/article/10.3390/ma17163951/s1, Preferred Reporting Items for Systematic Reviews and Meta-Analyses extension for Scoping Reviews (PRISMA-ScR) Checklist.
